# Aesthetic Rhinoplasty: Technique, 3-Dimensional Simulation, and Outcome Assessment

**DOI:** 10.1093/asjof/ojaa054

**Published:** 2020-12-04

**Authors:** Kitae E Park, Navid Pourtaheri, Seija Maniskas, Omar Allam, Derek M Steinbacher

**Affiliations:** 1 Section of Plastic and Reconstructive Surgery, Department of Surgery, Yale School of Medicine, New Haven, CT, USA; 2 Yale Plastic Surgery, New Haven, CT, USA

## Abstract

Communication of goals and realistic expectations between the surgeon and patient is a crucial step of aesthetic rhinoplasty. Three-dimensional (3D) imaging technology allows for sharing of simulated outcomes in the office setting, thereby facilitating this process. This article highlights the use of 3D rhinoplasty simulation in preoperative assessment and the senior author’s preferred surgical technique in open rhinoplasty.

Rhinoplasty can improve the appearance of the nose and its harmony with facial structures while optimizing nasal airflow. Common indications for rhinoplasty include congenital nasal deformity (ie, cleft nasal deformity), facial/nasal trauma, dissatisfaction with nasal aesthetics (ie, wide nasal bones and dorsal hump), and difficulty breathing (ie, nasal vestibular stenosis and deviated septum).

Various techniques have been described to aid in preoperative planning. Three-dimensional (3D) rhinoplasty simulation is a useful adjunct tool that can aid in operative planning and communication of goals between the patient and surgeon. In this article, we demonstrate the use of preoperative 3D rhinoplasty simulation and the technical aspects of open rhinoplasty in a patient performed by the senior author.

## INDICATIONS

The patient is a 30-year-old woman who presented with dissatisfaction with nasal appearance and intermittent breathing difficulty. Physical examination revealed a low radix, dorsal hump, midvault deviation, over-projected nasal tip, slit-like nostrils, and S-shaped deviation of the septum. Inferior turbinates were enlarged bilaterally. An open rhinoplasty approach was planned.

## PREOPERATIVE PLANNING

Preoperative 3D photogrammetry and rhinoplasty simulation were performed. Aspects of the surgery were simulated and discussed with the patient, including adjustment of tip projection, improvement of dorsal contour, and creation of a supratip break.

## OPERATIVE TECHNIQUE

The patient was brought to the operating room and prepped in a sterile fashion following anesthesia induction. A transcolumellar incision was performed, followed by exposure of the domes and midvault. A limited cephalic trim was performed bilaterally. The septum was accessed, and the upper lateral cartilages separated.

The nasal dorsum was lowered using component takedown techniques. Dorsal irregularities were then rasped. The caudal septum was trimmed, and a septoplasty was performed leaving a strong L-strut. Endonasal high-low-high osteotomies were completed. The dorsal upper lateral cartilage margins were carved into 2 autospreader flaps. The spreader flaps and graft were placed into position. Bilateral turbinates were outfractured and coblated. A septal extension graft was placed on the right-hand side, and the tip position was secured. An infralobular tip graft was placed as well. Two articulating alar rim grafts were placed, and the medial crural feet were secured.

Fat was harvested from the abdomen and processed by Telfa-rolling.^1^ The fat was then mixed with strips of crushed septal cartilage and the combination used as a dorsal onlay graft. Fat was also injected into the sidewall cheek border to alleviate bruising and swelling at the osteotomy sites.^2^ Kenalog was injected into the inferior turbinates, medial crural footplates, and medial canthal regions to reduce swelling. Incision sites were closed and dressing was placed. Further details on the intraoperative technique can be found in the video content (Video 1). 

## POSTOPERATIVE CARE

**Figure 1.**
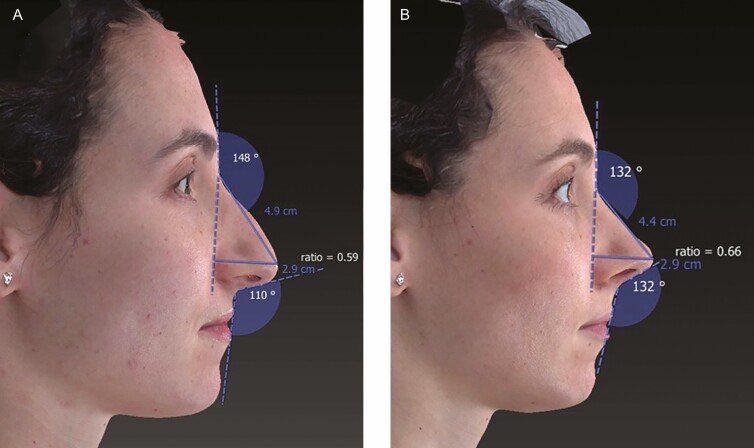
Comparison of nasofrontal angle, nasolabial angle, and Goode’s ratio preoperative (A) and 10 weeks postoperative (B) in a 30-year-old female patient who received open rhinoplasty.

The patient was seen for follow-up suture and splint removal at 1 week, and then regular intervals afterward. Typical postoperative care and recommendations were employed. Follow-up images in the video content were provided from 10 weeks postoperative. Three-dimensional analysis demonstrates improved Goode’s ratio, nasolabial angle, and nasofrontal angle postoperatively (Figure 1).

## DISCUSSION

Three-dimensional rhinoplasty simulation can facilitate communication between the surgeon and patient and help ensure that goals are aligned between both parties. In this process, it is important for the surgeon to display simulated results that are biologically attainable. Proficiency in the technique allows for consistent production of results that do not exceed the actual outcome, thereby preventing false expectations by the patient.^3,4^ Requests by the patient to perform multiple modifications or excessive changes on simulation may suggest an underlying body dysmorphic disorder that could contribute to persistent dissatisfaction with appearance following surgery.^5^ Limitations of 3D simulation include setup costs (equipment and software), pixilation, and simulation limitations on certain views (eg, frontal and submental). Three-dimensional technology involving radiography rather than 3D photogrammetry may involve radiation risk to the patient.

A variety of rhinoplasty maneuvers were employed to improve nasal appearance in the presented patient. The high nasal dorsum and tension tip, as well as a short upper lip, were among the patient’s most distressing complaints. A caudal septal trim, tongue-in-groove, and dissection to the anterior nasal spine were performed to correct the tension tip, lip length, and aesthetics at subnasale.^6,7^ Several grafts were used to support the tripod complex. The septal extension graft is a powerful technique that improves tip projection and rotation by serving as a secure point of the tripod.^8^ The graft can create a supratip break when placed at a step off (dorsally) from the caudal edge of the septum. The articulated alar rim graft is a modified form of the traditional alar rim contour graft that is sutured to the tip complex, rather than free floating in a soft tissue pocket.^9^ This technique improves upon the possibility of a shrink wrap-like contracture that may occur in the traditional alar rim graft.^10^ When secured to the caudal septal extension graft and lateral crura, the articulated alar rim graft provides strength to the tripod complex as displayed in the video.

Crushed autologous cartilage dorsal onlay grafts are a relatively new technique associated with improved dorsal irregularities and contour and high patient satisfaction.^11^ Septal cartilage is often used in crushed cartilage grafts due to its ease of harvesting during rhinoplasty. When mixed with prepared fat as performed by the senior author, the adipose tissue may serve as a scaffold for cartilage placement as well as further smoothen minor defects in dorsal contour. A limitation of this technique is related to patients with a paucity of septal cartilage, as in the case of revision rhinoplasty patients.

Finally, harvested fat was injected into the sidewall cheek border/osteotomy sites. This has been shown to reduce postoperative edema and ecchymosis following open rhinoplasty.^2^

## CONCLUSIONS

Preoperative 3D rhinoplasty simulation can foster communication of goals and expectations between the patient and surgeon. The video demonstrates modern rhinoplasty maneuvers, including caudal septal trim, septal extension graft, and articulating alar rim grafts. Crushed cartilage mixed with autologous fat is introduced as an approach to smooth and augment nasal dorsal contour. Autologous fat injections are also used, which has been shown to reduce postoperative edema and ecchymosis and enhance the aesthetic result.
